# Discovery of Novel Small Molecule Activators of β-Catenin Signaling

**DOI:** 10.1371/journal.pone.0019185

**Published:** 2011-04-29

**Authors:** Folkert Verkaar, Mario van der Stelt, W. Matthijs Blankesteijn, Antoon A. van der Doelen, Guido J. R. Zaman

**Affiliations:** 1 Department of Molecular Pharmacology, Merck Research Laboratories, Oss, The Netherlands; 2 Department of Pharmacology and Toxicology, Maastricht University, Maastricht, The Netherlands; 3 Department of Medicinal Chemistry, Merck Research Laboratories, Oss, The Netherlands; Northwestern University Feinberg School of Medicine, United States of America

## Abstract

Wnt/β-catenin signaling plays a major role in embryonic development and adult stem cell maintenance. Reduced activation of the Wnt/β-catenin pathway underlies neurodegenerative disorders and aberrations in bone formation. Screening of a small molecule compound library with a β-galactosidase fragment complementation assay measuring β-catenin nuclear entry revealed bona fide activators of β-catenin signaling. The compounds stabilized cytoplasmic β-catenin and activated β–catenin-dependent reporter gene activity. Although the mechanism through which the compounds activate β-catenin signaling has yet to be determined, several key regulators of Wnt/β-catenin signaling, including glycogen synthase kinase 3 and Frizzled receptors, were excluded as the molecular target. The compounds displayed remarkable selectivity, as they only induced β-catenin signaling in a human osteosarcoma U2OS cell line and not in a variety of other cell lines examined. Our data indicate that differences in cellular Wnt/β-catenin signaling machinery can be exploited to identify cell type-specific activators of Wnt/β-catenin signaling.

## Introduction

Wnt/β-catenin signaling orchestrates embryogenesis and adult stem cell maintenance in mammals [1]. It is initiated when Wnt ligands bind to seven transmembrane receptors of the Frizzled family and to representatives of the single-pass low-density lipoprotein receptor-related protein family (LRP5 or -6) [2], [3], [4]. Wnt, Frizzled and LRP5/6 form a ternary complex that initiates a cascade of molecular interactions that ultimately leads to the cytoplasmic stabilization of the transcriptional modulator β-catenin. β-Catenin subsequently enters the nucleus where it interacts with T cell factor/Lymphoid enhancer factor (TCF/LEF) and influences the transcription of β-catenin-dependent genes [5]. In non-stimulated cells, β-catenin protein stability is compromised by the glycogen synthase kinase 3 (GSK3)-mediated phosphorylation of β-catenin on several conserved N-terminal residues. These phosphorylations serve as cues for proteasomal degradation of β-catenin. As a result, quiescent cells typically contain low levels of cytoplasmic and nuclear β-catenin.

Aberrations in Wnt/β-catenin signaling activity are associated with several malignancies [6]. For example, mutations that lead to increased β-catenin stability are observed in the large majority of colon cancers [6], and drug discovery efforts have mainly focused on identifying inhibitors for the β-catenin pathway [7]. The potential medical application of activators of β-catenin signaling has been largely overlooked, despite evidence that reduced β-catenin signaling underlies neurodegenerative disorders and aberrations in bone formation [8], [9]. We have previously described a cell-based assay that measures the nuclear translocation of β-catenin using enzyme fragment complementation (EFC) [10]. In this assay, complementation occurs between a peptide fragment of β-galactosidase (called α-peptide) that is genetically fused to β-catenin and a nuclear-resident complementary enzyme fragment (termed Δα-Nuc). By applying this assay to screening of a low molecular weight compound library, we have identified novel activators of β-catenin signaling.

## Results

**Figure 1:**
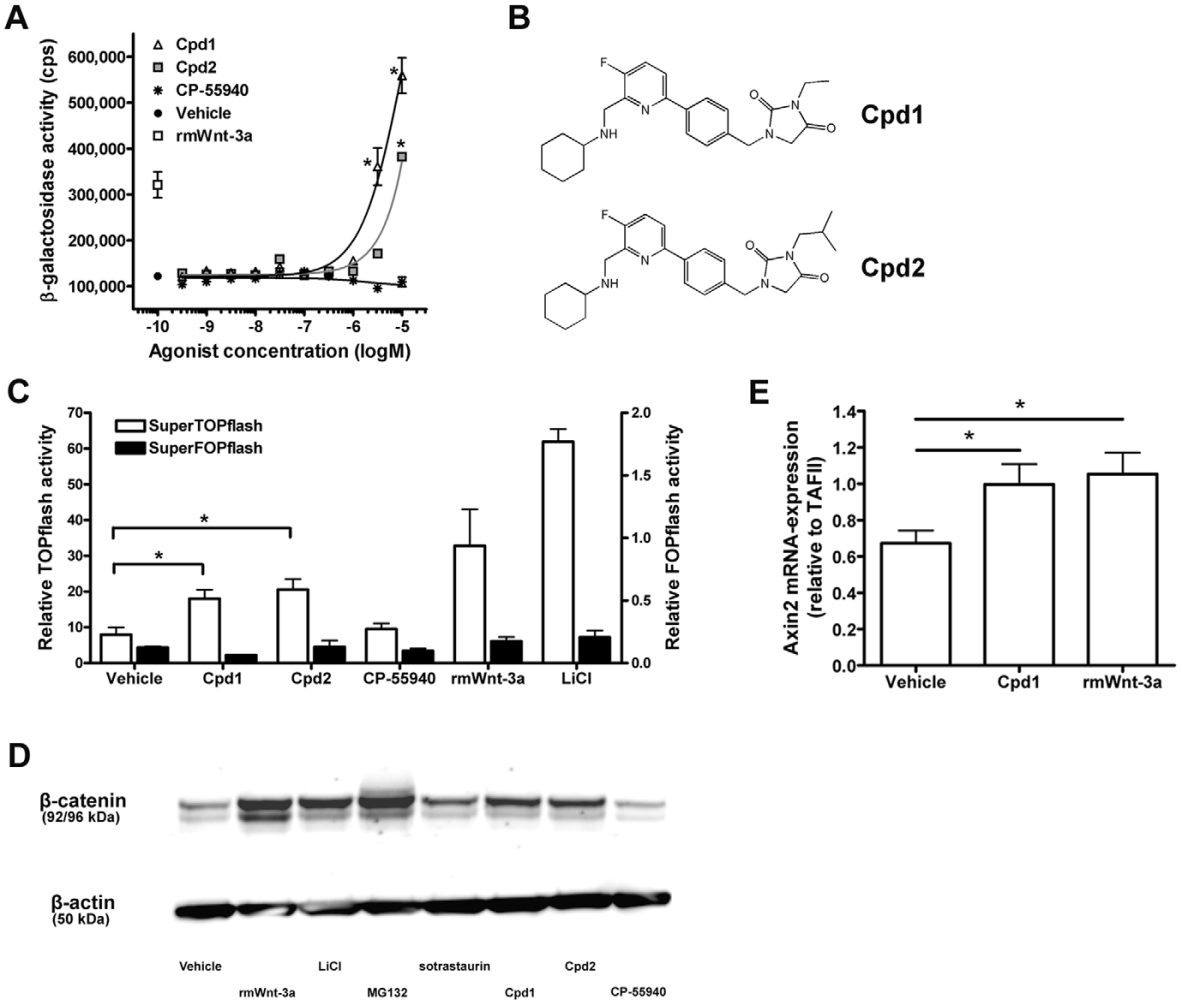
Cpd1 and Cpd2 activate Wnt/β-catenin signaling in U2OS-EFC cells. (A) U2OS-EFC cells were treated with vehicle, 12 nM rmWnt-3a or increasing concentrations of Cpd1, Cpd2 and CP-55940 for 3 hrs, followed by measurement of β-galactosidase activity. (B) Structures of Cpd1 and Cpd2. (C) U2OS-EFC cells transiently transfected with SuperTOPflash (white bars) or SuperFOPflash (black bars) reporter gene construct were incubated with vehicle, 12 nM rmWnt-3a, 30 mM lithium chloride (LiCl) or 10 µM Cpd1, Cpd2 or CP-55940 for 5 hrs before measurement of luciferase activity. (D) U2OS-EFC cells were treated with vehicle, 12 nM rmWnt-3a, 30 mM LiCl, 20 µM MG132 or 10 µM sotrastaurin, Cpd1, Cpd2 or CP-55940 for 3 hrs. Cytoplasmic protein fractions were isolated and subjected to western blotting for β-catenin and β-actin. (E) U2OS-EFC cells were treated with vehicle, 12 nM rmWnt-3a or 10 µM Cpd1 for 8 hrs before analysis of axin2 mRNA levels relative to the expression of the housekeeping gene TATA box-binding protein (TAFII) by means of qPCR. Asterisks (*) represent statistically significant differences (P<0.05).

We tested 2300 drug-like compounds with activity towards G protein-coupled receptors (GPCR) or kinases for their ability to activate Wnt/β-catenin signaling in a human osteosarcoma U2OS cell line (termed U2OS-EFC) genetically engineered to couple nuclear entry of β-catenin to increases in β-galactosidase complementation [10] and found three hits. One of these was sotrastaurin (AEB-071), an anti-inflammatory drug that was found to inhibit GSK3 [10]. The other two hits, denoted Cpd1 and Cpd2 (Figure 1A), did not inhibit GSK3 or any other kinase in a panel of more than 200 kinases tested at 10 µM in biochemical assays (performed at Millipore; data not shown). Cpd 1 and 2 (Figure 1B) have been described previously as cannabinoid CB2 receptor agonists in a patent application (WO2010/063666) [11]. To investigate whether the cannabinoid CB2 receptor was involved in the activation of Wnt/β-catenin signaling, reference cannabinoid CB1/2 agonists CP-55940, WIN55,212-2, and several structural analogs of Cpd1 and Cpd2 were tested in the assay. However, none of these potent cannabinoid CB2 agonists exerted any significant effect (Figure 1A), indicating that the effect was compound specific and activation of the cannabinoid CB2 receptors was not the mechanism through which Cpd1 and Cpd2 increased β–galactosidase activity in the assay.

At a 10 µM concentration, both Cpd1 and Cpd2 induced transcription of a transiently transfected SuperTOPflash reporter gene in U2OS-EFC cells (Figure 1C). We observed micromolar potencies for both compounds (data not shown), similar to those observed in the EFC assay (Figure 1A). As controls, we treated these cells with 12 nM recombinant mouse Wnt-3a (rmWnt-3a) and 30 mM lithium chloride (LiCl) (Figure 1C). Wnt-3a is a naturally occurring Wnt ligand that activates Wnt/β-catenin signaling in several contexts [12], [13]. LiCl and MG132 (see below) inhibit GSK3 and the proteasome, respectively. Treating cells with these agents causes β-catenin stabilization and subsequent activation of Wnt/β-catenin signaling [10]. Cpd1 and Cpd2 did not induce transcription of a mutant luciferase reporter gene construct containing β-catenin-responsive elements that do not bind TCF (SuperFOPflash; Figure 1C), demonstrating selectivity of the response. We subsequently studied the cytoplasmic accumulation of β-catenin in U2OS-EFC cells following a three hour treatment with rmWnt-3a, LiCl, sotrastaurin and MG132. As predicted, these treatments resulted in an increase in cytoplasmic levels of both non-modified (92 kDa) and α-peptide-tagged (96 kDa) β-catenin, as assessed by Western blotting (Figure 1D). Cpd1 and Cpd2 also stabilized cytoplasmic β-catenin, whereas CP-55940 did not (Figure 1D). Finally, qPCR analysis showed that the mRNA levels of the β-catenin-responsive gene axin2 [14], [15] were up-regulated by treatment with Cpd1 in U2OS-EFC cells (Figure 1E) and in U2OS-Δα-Nuc cells (Figure S1), which are the parental cells of U2OS-EFC cells.

**Figure 2:**
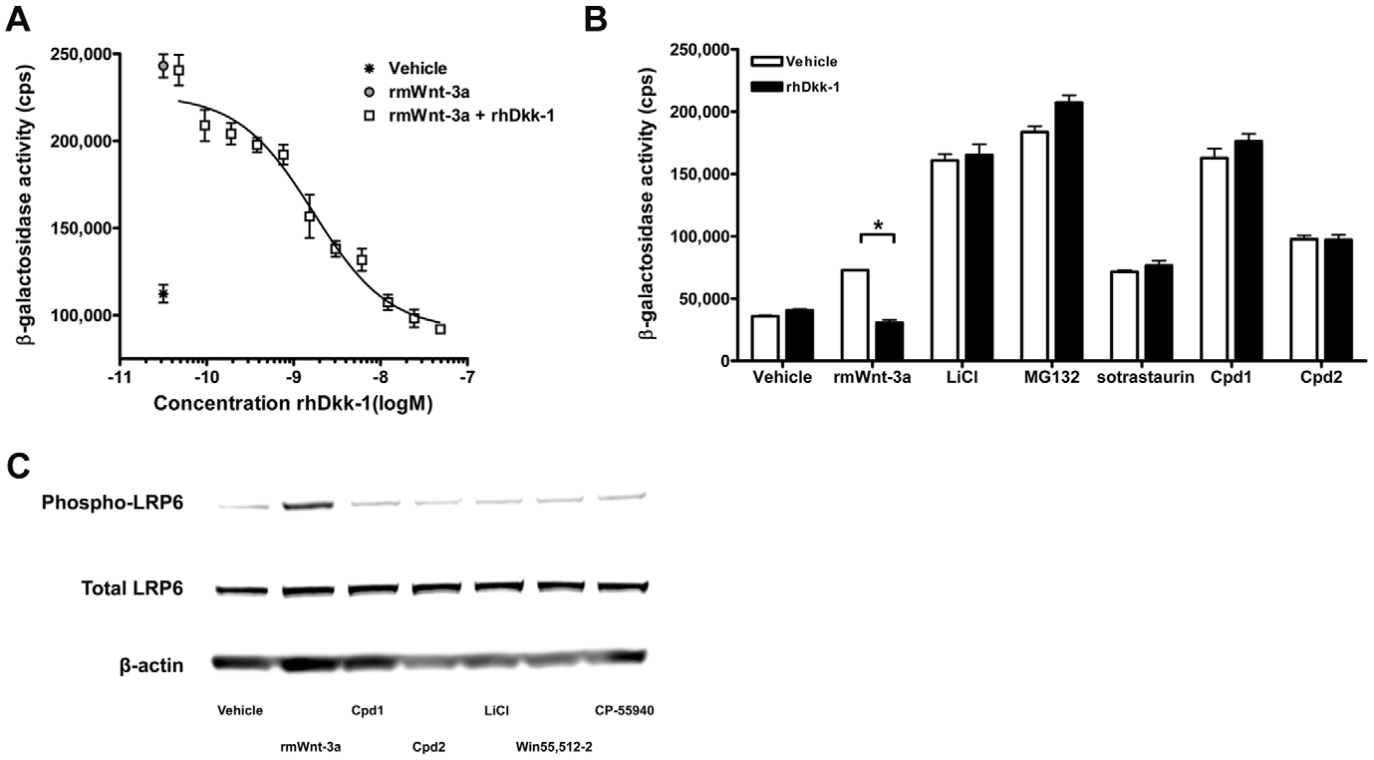
Cpd1 and Cpd2 do not activate Frizzleds. (A) U2OS-EFC cells were treated with vehicle or 10 nM rmWnt-3a in the absence or presence of increasing concentrations of rhDkk-1 for 3 hrs, followed by measurement of β-galactosidase activity. (B) U2OS-EFC cells were stimulated with 10 nM rmWnt-3a, 30 mM LiCl, 20 µM MG132 or 10 µM sotrastaurin, Cpd1 and Cpd2 in the presence of absence of 48 nM rhDkk-1 for 3 hrs, after which β-galactosidase activities were measured. (C) U2OS-EFC cells were treated with vehicle, 12 nM rmWnt-3a, 30 mM LiCl or 10 µM Cpd1, Cpd2, Win55,512-2 and CP-55940 for 1 hr, followed by cell lysis and western blotting for β-actin, total LRP6 and LRP6 phosphorylated at Ser1490. Asterisks (*) represent statistically significant differences (P<0.05).

Because Frizzleds are structurally related to GPCRs, we investigated whether Cpd1 and Cpd2 are capable of activating Frizzleds. To assess this, we used recombinant human Dickkopf-1 (rhDkk-1) protein, which recruits LRP5/6 to the membrane protein Kremen, leading to internalization of LRP5/6 [16], [17]. This causes cells to become unresponsive to Wnts. We confirmed desensitization by incubating U2OS-EFC cells with increasing concentrations of rhDkk-1 in the presence of 10 nM rmWnt-3a. As can be seen in Figure 2A, rhDkk-1 dose-dependently decreased β-catenin signaling induced by rmWnt-3a. However, 2 µg/ml rhDkk-1 did not reduce β-galactosidase activity induced by Cpd1 or Cpd2, whereas it reduced rmWnt-3a-induced signaling to background levels (Figure 2B). Furthermore, Cpd1 and Cpd2 did not induce phosphorylation of LRP6 at Ser1490 (Figure 2C), an early event during Wnt/β-catenin pathway activation [18], [19]. This suggests that both compounds interact with Wnt/β-catenin signaling at a level between LRP5/6 receptor activation and cytoplasmic accumulation of β-catenin.

**Figure 3:**
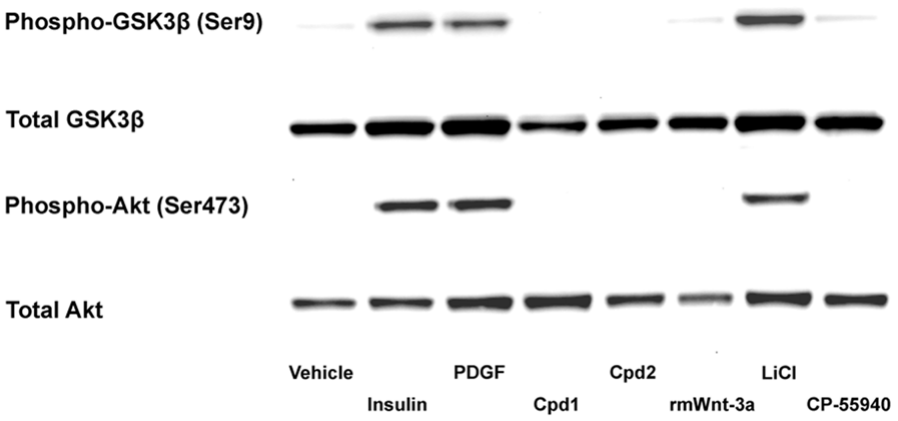
Cpd1 and Cpd2 do not cause activation of Akt/PKB signaling in U2OS-EFC cells. U2OS-EFC cells were stimulated with vehicle, 100 nM insulin, 100 ng/ml platelet-derived growth factor (PDGF), 12 nM rmWnt-3a, 30 mM LiCl or 10 µM Cpd1, Cpd2 and CP-55940 for 30 min, followed by cell lysis. Akt/protein kinase B (PKB) and glycogen synthase kinase 3β (GSK3β) phosphorylations at Ser473 and Ser9, respectively, were analyzed by western blotting.

Although biochemical profiling indicated that Cpd1 or Cpd2 did not act through inhibition of GSK3 or any other protein kinase, the possibility that the compounds inhibit GSK3 activity by inducing phosphorylation of an N-terminal serine residue (Ser21 in GSK3α and Ser9 in GSK3β) can only be verified in cells. Phosphorylation of GSK3β at Ser9 is catalyzed by protein kinase B (PKB)/Akt, when cells are stimulated with insulin or platelet-derived growth factor (PDGF) [20]. To investigate whether Cpd1 and Cpd2 could induce GSK3β phosphorylation at Ser9, U2OS-EFC cells were treated for 30 min with 100 nM insulin, 100 ng/ml PDGF, or 10 µM Cpd1 or Cpd2. As is apparent from Figure 3, insulin and PDGF induced a marked increase in the phosphorylation of PKB/Akt at Ser473 and of GSK3β at Ser9. However, Cpd1 and Cpd2 did not induce PKB/Akt or GSK3β phosphorylation (Figure 3). These results suggest that Cpd1 and Cpd2 do not affect GSK3 function. Thus, our data demonstrate that Cpd1 and Cpd2 activate Wnt/β-catenin signaling upstream of β-catenin stabilization and downstream of LRP5/6 phosphorylation (Figure S2).

696 structural analogs of Cpd1 and Cpd2 were selected from the Merck research Laboratories compound library and tested in the β-catenin EFC assay. 18 compounds were found to increase β-galactosidase activity at 10 µM and two compounds (Cpd4 and 5 in Figure S3) were even more efficacious than Cpd1. There was no apparent correlation between biophysical parameters, such as solubility or lipophylicity, and activity in the β-catenin EFC assay (data not shown), indicating that the activity in the assay was through interaction with a specific target. We have attempted to identify the target by profiling Cpd1 against 80 GPCR-, transporter- and ion channel targets (performed at Cerep, Paris, France) and 21 human phosphatases (performed at Millipore), but these screens did not reveal any specific interactions that could account for the activating effect of Cpd1 on β-catenin signaling in our cell-based assays.

**Figure 4:**
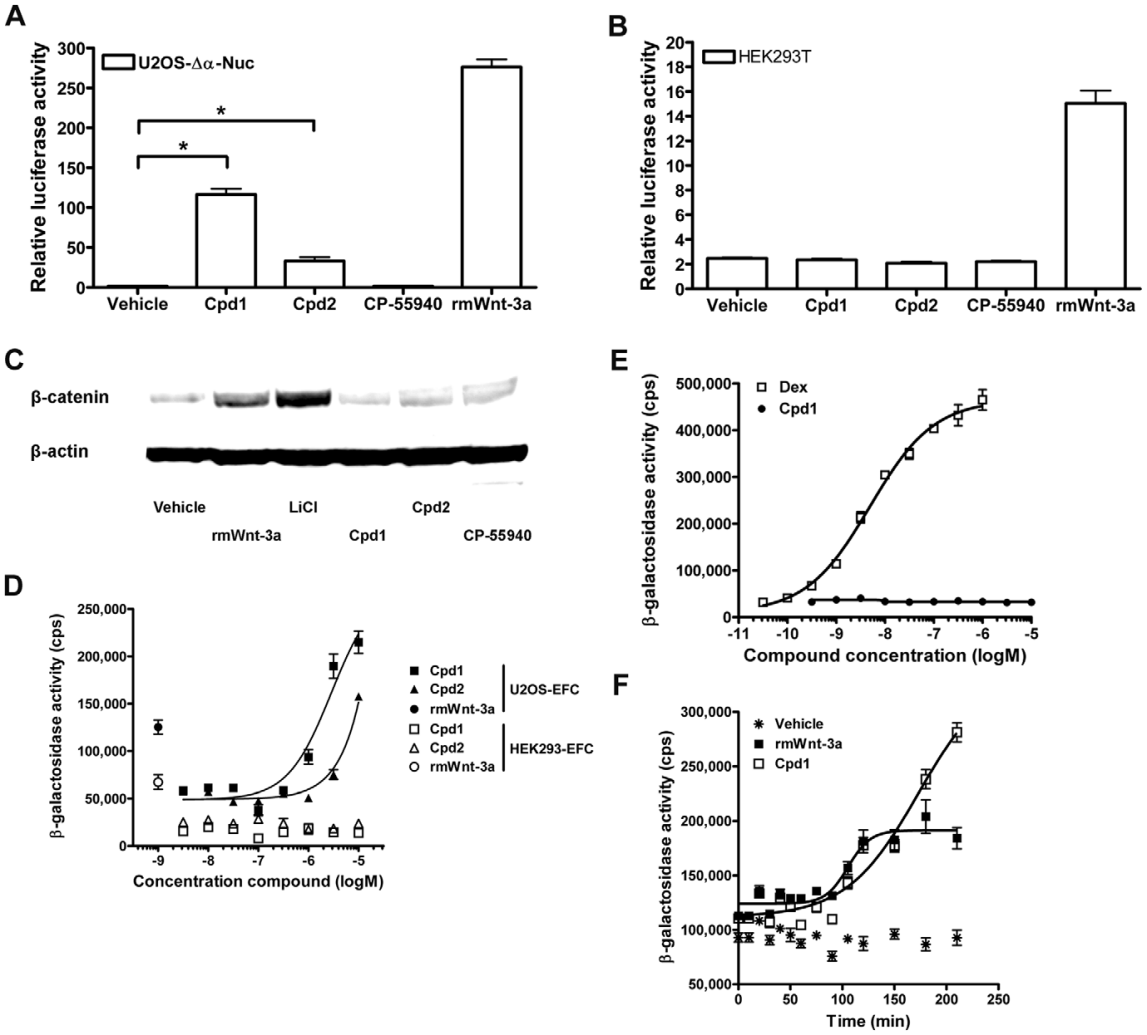
Cpd1 and Cpd2 do not activate Wnt/β-catenin signaling in HEK293 cells. (A,B) U2OS-Δα-Nuc cells (parental to U2OS-EFC cells) (A) and HEK293T cells (B) transiently transfected with SuperTOPflash reporter gene construct were stimulated with 12 nM rmWnt-3a or 10 µM Cpd1, Cpd2 and CP-55940 for 5 hrs followed by measurement of luciferase activity. (C) HEK293T cells were stimulated with vehicle, 12 nM rmWnt-3a, 30 mM LiCl or 10 µM Cpd1, Cpd2 and CP-55940 for 3 hrs, followed by isolation of cytoplasmic protein fractions and probing of β-catenin and β-actin using western blotting. (D) U2OS-EFC cells and HEK293 cells genetically engineered to complement β-galactosidase upon nuclear translocation of β-catenin (HEK293-EFC cells) were stimulated with increasing concentrations of Cpd1 and Cpd2 or 12 nM rmWnt-3a for 3 hrs, followed by measurement of β-galactosidase activity. (E) U2OS-Δα-Nuc cells stably transfected with a vector coding for α-peptide-tagged human glucocorticoid receptor (U2OS-GR cells) were treated with increasing doses of dexamethasone (Dex; a GR agonist) or Cpd1 for 3 hrs before measurement of β-galactosidase activity. (F) U2OS-EFC cells were stimulated with 12 nM rmWnt-3a or 10 µM Cpd1 for several time points before measurement of β-galactosidase activity.

We extended our analysis of Cpd1/2 to different cell lines by transient transfection of superTOPflash reporter gene construct, followed by treatment with compound. As expected, U2OS-Δα-Nuc cells responded to Cpd1 and Cpd2 with a marked increase in reporter gene activity (Figure 4A). In contrast, the superTOPflash reporter was not activated in human embryonic kidney (HEK293T) cells, cervical cancer (HeLa) cells or Chinese hamster ovary (CHO-K1) cells after Cpd1/2 treatment (Figure 4B, Figure S4A,B). Consistent with these findings, cytoplasmic β-catenin levels of HEK293T cells were not increased by treatment with 10 µM Cpd1 or Cpd2 (Figure 4C). Notably, Cpd1 and Cpd2 did not activate reporter gene activity in another human osteosarcoma cell line (Saos2), or in a rat osteosarcoma (UMR106) cell line (Figure S4C,D). Most strikingly, the compounds were inactive in a U2OS cell batch directly obtained from the American Type Culture Collection (ATCC; Figure S4E). We confirmed that the U2OS-EFC cells shared a common ancestry with the U2OS cells from ATCC by microsatellite analysis, although the analysis indicated that genetic drift had occurred (performed at Bioreliance, Glasgow, UK; data not shown).

Despite the absence of β-catenin activation in any cell line other than U2OS-EFC cells and U2OS-Δα-Nuc cells, our data do not suggest that activation of β-galactosidase activity by Cpd1 and Cpd2 is an assay artifact. Firstly, other parameters of Wnt/β-catenin signaling, such as β-catenin-dependent reporter genes and Western blotting of cytoplasmic β-catenin levels also imply activation of Wnt/β-catenin signaling in these cells (Figure 1C–E, Figure 4A). As an additional control experiment, we transfected U2OS-Δα-Nuc cells with luciferase reporter genes sensitive to cAMP (CREB), Ca^2+^ (NFAT) and glucocorticoid hormone receptor (GR) signaling. None of these reporter genes was activated by Cpd1, whereas SuperTOPflash reporter gene activity was dose-dependently induced by Cpd1 treatment (Figure S5A–D). Furthermore, HEK293 cells engineered to complement β-galactosidase in response to rmWnt-3a (HEK293-EFC cells) did not activate β-catenin signaling when treated with Cpd1 or Cpd2 (Figure 4D). In addition, Cpd1 did not induce β-galactosidase activity in a cellular EFC assay for the recruitment of the scaffolding protein β-arrestin2 to human parathyroid hormone receptor 1 (Figure S6). Furthermore, Cpd1 did not induce the nuclear translocation of human GR in U2OS cells (U2OS-GR) genetically engineered to complement β-galactosidase in response to GR agonists [10], such as dexamethasone (Dex; Figure 4E). Of note, U2OS-GR cells are derivatives of the U2OS-Δα-Nuc cell line. Lastly, time-course EFC experiments revealed that the onset of signal generation in U2OS-EFC cells stimulated with 10 µM Cpd1 is similar to that generated in U2OS-EFC cells treated with 500 ng/ml rmWnt-3a (Figure 4F), although β-galactosidase activity in response to rmWnt-3a seems to reach a plateau earlier. A signal generation profile similar to that seen for Cpd1 was observed when U2OS-EFC cells were treated with 30 mM LiCl (Figure S7). All these experiments indicate that Cpd1/2's ability to activate Wnt/β-catenin is a highly specific, cell-type dependent process.

## Discussion

Activation of the Wnt/β-catenin pathway might provide a new therapeutic opportunity to treat neurodegenerative disorders and aberrations in bone formation. Stimulation of the Wnt/β-catenin pathway by compounds only in specific tissues is expected to generate a better side-effect profile. We have identified small molecule activators of Wnt/β-catenin signaling in a U2OS cell line that did not activate this pathway in various other cell types from different histogenic origin. The molecular target through which the compounds activate β-catenin signaling has yet to be determined, although several key regulators of β-catenin signaling, including GSK3 and Frizzled receptors, were excluded.

Integrative approaches coupling protein interaction maps to siRNA screening data have suggested that the components that constitute the Wnt/β-catenin signaling machinery in a given cell type are highly variable [21]. Our data confirm that small molecule-mediated cell-type specific activation of Wnt/β-catenin signaling can be achieved. However, elucidation of the molecular target is essential to fully appreciate this finding, and is desirable before these compounds are considered as a starting point for drug discovery. A possible strategy for target identification is biotin-labeling, followed by affinity capture of binding partners in cell lysates. However, such approaches are generally more successful with compounds that bind to their target with high affinity, while screening of several hundreds of analogs did not reveal compounds with potencies lower than 1 µM.

In conclusion, we have identified small molecule compounds that activate Wnt/β-catenin signaling in a highly cell-type specific manner. Our data hold promise for the development of tissue-specific β-catenin signaling activators.

## Materials and Methods

### Cell lines

HEK293T, U2OS, CHO-K1, LM-TK and HeLa cells were obtained from the American Type Culture Collection (ATCC) and cultured in DMEM F12 containing 10% fetal bovine serum (FBS; Cambrex, Verviers, Belgium), 100 U/ml penicillin and 100 µg/ml streptomycin (Invitrogen, Breda, The Netherlands). U2OS-Δα-Nuc cells (DiscoveRx; Hannover, Germany) were maintained in the same medium, supplemented with 150 µg/ml hygromycin (Invitrogen). U2OS-EFC, U2OS-GR and HEK293-EFC cells, the generation of which is described elsewhere [10], were cultured in DMEM F12 supplemented with 10% FBS, 100 U/ml penicillin, 100 µg/ml streptomycin, 150 µg/ml hygromycin and 500 µg/ml geneticin (Invitrogen).

### LMW compounds and recombinant proteins

Synthetic organic low-molecular weight (LMW) compounds were selected from the Merck Research Laboratories compound collection (Oss, The Netherlands) based on previously determined activity on various G protein-coupled receptor (GPCR) or protein kinase targets. Recombinant mouse Wnt-3a (rmWnt-3a), recombinant human Wnt inhibitory factor-1 (WIF1) and recombinant human Dickkopf-1 (rhDkk-1) were purchased from R&D Systems (Abingdon, U.K.). Platelet-derived growth factor (PDGF) was obtained from PeproTech (Rocky Hill, NJ).

### β-Galactosidase fragment complementation assays

The β-catenin EFC assay was performed as described previously [10].

### Reporter gene assays

Luciferase assays on transiently transfected cells were performed as described before [10]. The pTA-SuperTOPflash and pTA-SuperFOPflash reporter gene constructs were provided by Prof. dr. R.T Moon (University of Washington, WA, USA). p21xCRE-luc was obtained from the VU University (Amsterdam, The Netherlands). pTA-NFAT-luc was purchased from Clontech. pMMTV-luc and pNGV1-GR were provided by Hans van der Maaden (Merck Research Laboratories). For MMTV-luciferase reporter gene experiments, pMMTV-luc and pNGV1-GR were co-transfected in a 5∶1 molar ratio.

### Western blotting

Isolation of cytoplasmic fractions and total cell lysates, followed by western blotting, was described previously [10]. The following antibodies were used at the indicated dilutions: mouse anti-β-catenin: 1∶2000 (BD Transduction Laboratories; Lexington, KY), mouse anti-β-actin: 1∶5000 (Abcam; Cambridge, UK), rabbit-anti-Akt: 1∶1000, rabbit anti-GSK3β: 1∶1000, rabbit anti-phospho-Ser473 Akt: 1∶1000, rabbit anti-phospho-Ser9 GSK3β: 1∶1000, rabbit anti-LRP6: 1∶1000 and rabbit anti-phospho-Ser1490 LRP6: 1∶1000 (Cell Signaling Technology; Danvers, MA), anti-mouse-HRP conjugate: 1∶2000 and anti-rabbit-HRP conjugate: 1∶1000 (Promega; Leiden, The Netherlands).

### qPCR analysis

Isolation of total mRNA and subsequent analysis of transcript levels by qPCR was performed as described previously [22].

### Data analysis

The concentration at which half-maximal activation of the cellular response was reached (EC_50_ values) for rhDkk1 and rmWnt-3a dose response curves was calculated from the expected molecular mass of rhDkk-1 and rmWnt-3a (both 41 kDa) using Graphpad Prism 4.0 software. All data are represented as averages ± standard error in the mean (SEM). Statistical significance of observed differences was determined using Student's t-test and indicated in the figures with asterisks (*). P<0.05 was regarded as statistically significant.

## Supporting Information

Figure S1 **Cpd1 induces transcription of the β-catenin-responsive gene axin2 in U2OS-Δα-Nuc cells.** U2OS-Δα-Nuc cells were treated with vehicle, 12 nM rmWnt-3a or 10 µM Cpd1 for 8 hrs before analysis of axin2 mRNA levels relative to the expression of the housekeeping gene TATA box-binding protein (TAFII) by means of qPCR. Asterisk represent statistically significant differences (p<0.05).
(TIF)

Figure S2 **Schematic representation of the mode of action of Cpd1 and Cpd2.** A model of Wnt/β-catenin signaling, in which activation of Frizzled receptors by Wnt-3a leads to subsequent phosphorylation of LRP5/6, inhibition of GSK3, β-catenin accumulation, β-catenin nuclear translocation and β-catenin-dependent gene transcription. Cpd1 and Cpd2 activate Wnt/β-catenin signaling at the level of β-catenin accumulation.
(TIF)

Figure S3 **Structural analogs of Cpd1 and Cpd2 activate Wnt/β-catenin signaling in U2OS-EFC cells.** U2OS-EFC cells were treated for 3 hrs with 10 nM rmWnt-3a or 10 µM of Cpd1-5, before measurement of β-galactosidase activity.
(TIF)

Figure S4 **Cpd1 and Cpd2 do not activate Wnt/β-catenin signaling in several commonly used cell lines from different origins.** HeLa (A), CHO-k1 (B), Saos-2 (C), UMR106 (D) and U2OS cells derived from ATCC (E) were transiently transfected with TOPflash reporter gene construct and stimulated with 10 µM Cpd1, Cpd2 or CP55940 or 12 nM rmWnt-3a for 5 hrs prior to measurement of luciferase activity.
(TIF)

Figure S5 **Cpd1 activates SuperTOPflash, but not CREB-, NFAT- and GR-dependent reporter gene activity in U2OS-Δα-Nuc cells.** U2OS-Δα-Nuc cells were transiently transfected with vectors encoding luciferase under the transcriptional control of (A) β-catenin (SuperTOPflash), (B) CREB (21xCRE-luc), (C) NFAT/Ca^2+^ (NFAT-luc) and (D) GR (MMTV-luc). Cells were stimulated with ascending concentrations of Cpd1 and reference agonists: (A) 10 nM rmWnt-3a, (B) 10 µM isoproterenol, an agonist for endogenously expressed Gs-coupled β2 adrenergic receptors, (C) a combination of 100 nM phorbol 12-myristate 13-acetate (PMA) and 100 nM thapsigargin (Thaps), which activate protein kinase C and Ca^2+^-signaling, respectively, and (D) 1 µM of the GR agonist dexamethasone (Dex).
(TIF)

Figure S6 **Cpd1 does not activate β-galactosidase activity in a CHO cell line genetically engineered to couple recruitment of the scaffolding protein β-arrestin2 to the human parathyroid hormone receptor 1 (hPTH1R).** CHO-PTH1R cells were stimulated with increasing concentrations of Cpd1 or with 100 nM human PTH1-34 for 90 min, followed by measurement of β-galactosidase activity.
(TIF)

Figure S7 **The increase in β-galactosidase activity in U2OS-EFC cells following treatment with Cpd1 has a profile that is similar to that observed for U2OS-EFC cells treated with LiCl.** U2OS-EFC cells were treated with 10 µM Cpd1 or 30 mM LiCl for several time periods before measurement of β-galactosidase activity.
(TIF)
