# Supplementary figures and images for: Discovery of Novel Small Molecule Activators of β-Catenin Signaling
Source: PLoS One. 2011 Apr 29;6(4):e19185. doi: 10.1371/journal.pone.0019185 (PMC3084789; doi:10.1371/journal.pone.0019185)

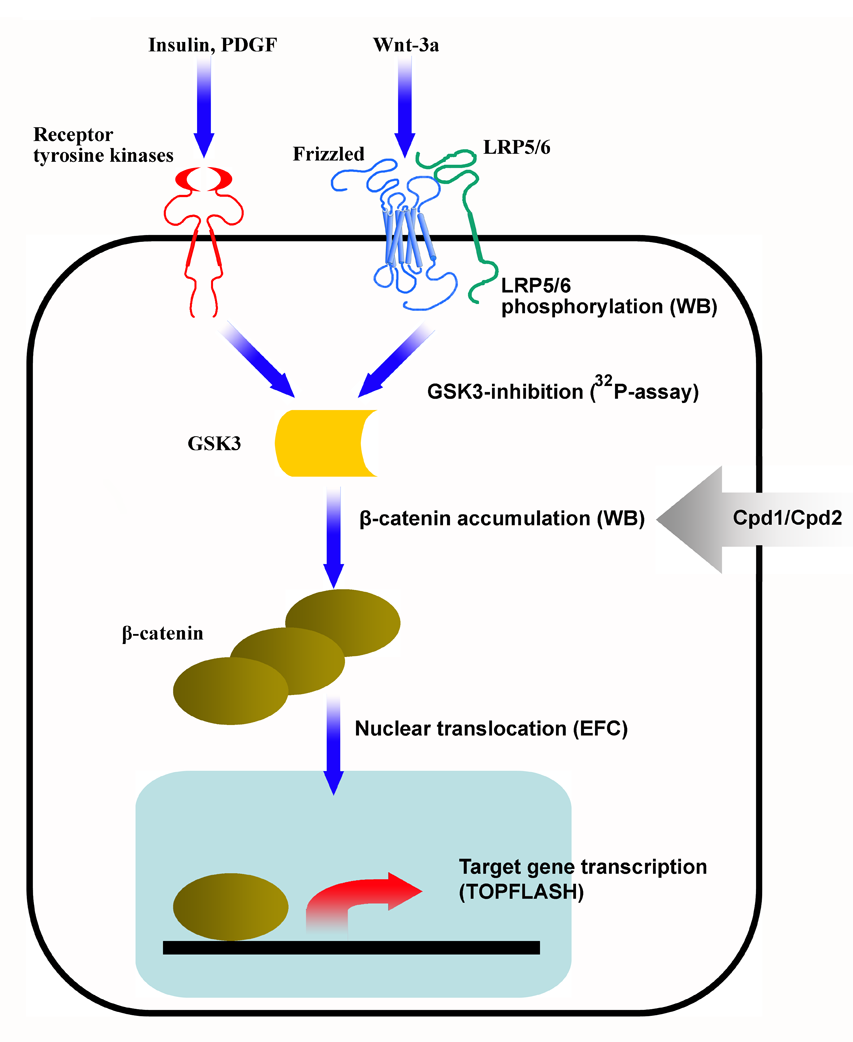

Supplement: Figure S2 — Schematic representation of the mode of action of Cpd1 and Cpd2. A model of Wnt/β-catenin signaling, in which activation of Frizzled receptors by Wnt-3a leads to subsequent phosphorylation of LRP5/6, inhibition of GSK3, β-catenin accumulation, β-catenin nuclear translocation and β-catenin-dependent gene transcription. Cpd1 and Cpd2 activate Wnt/β-catenin signaling at the level of β-catenin accumulation. (TIF) [file pone.0019185.s002.tif]

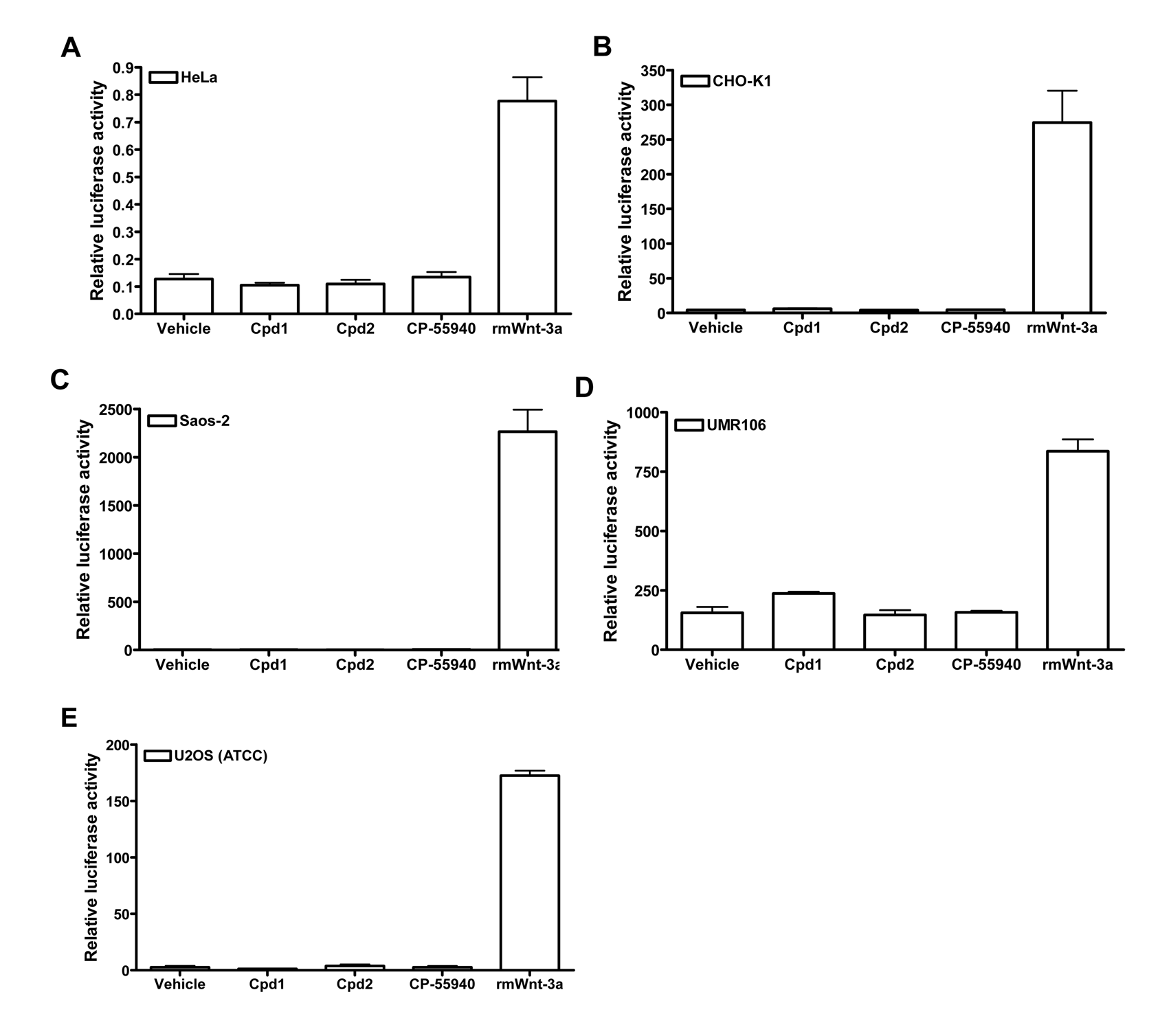

Supplement: Figure S4 — Cpd1 and Cpd2 do not activate Wnt/β-catenin signaling in several commonly used cell lines from different origins. HeLa (A), CHO-k1 (B), Saos-2 (C), UMR106 (D) and U2OS cells derived from ATCC (E) were transiently transfected with TOPflash reporter gene construct and stimulated with 10 µM Cpd1, Cpd2 or CP55940 or 12 nM rmWnt-3a for 5 hrs prior to measurement of luciferase activity. (TIF) [file pone.0019185.s004.tif]

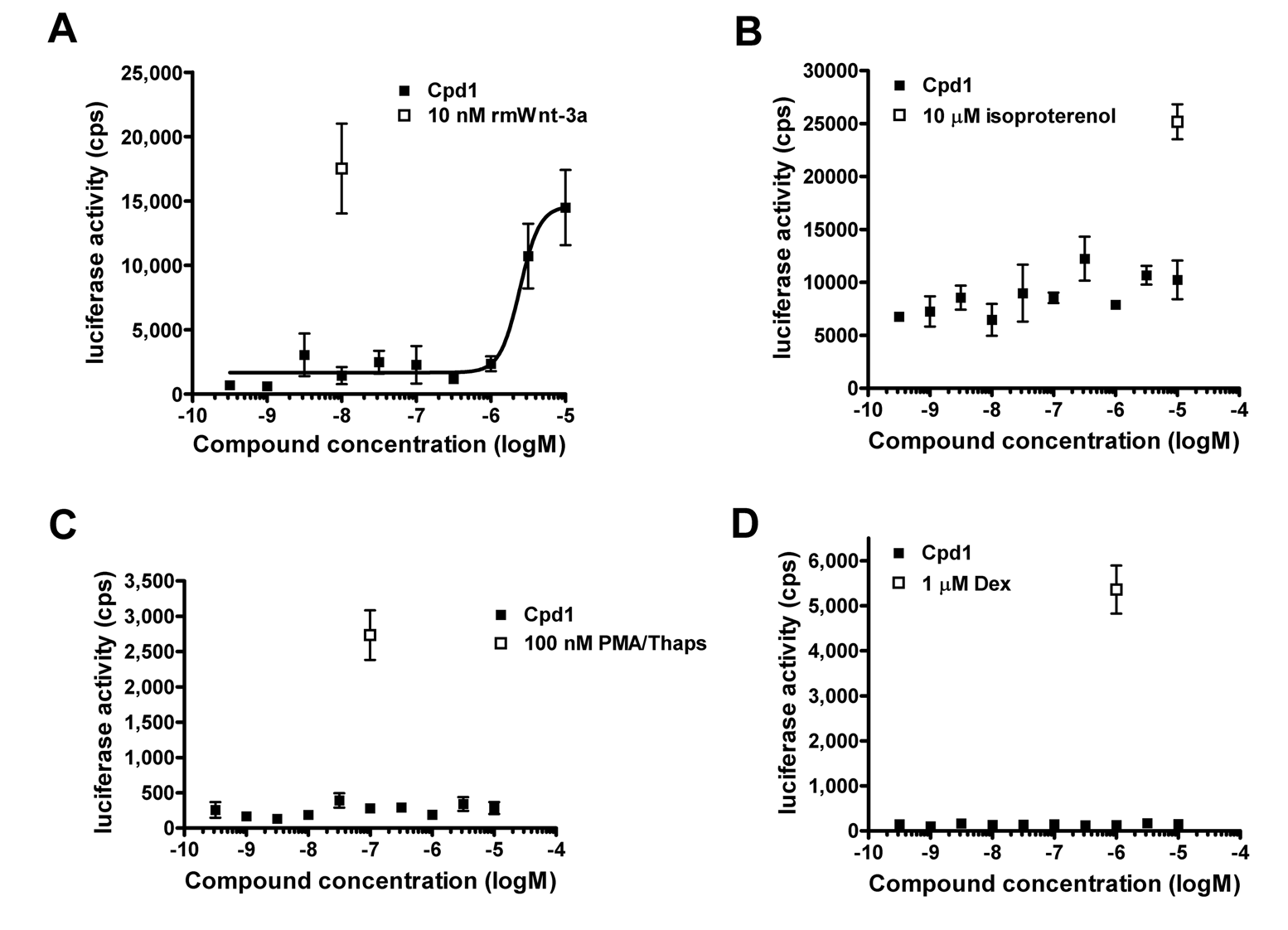

Supplement: Figure S5 — Cpd1 activates SuperTOPflash, but not CREB-, NFAT- and GR-dependent reporter gene activity in U2OS-Δα-Nuc cells. U2OS-Δα-Nuc cells were transiently transfected with vectors encoding luciferase under the transcriptional control of (A) β-catenin (SuperTOPflash), (B) CREB (21xCRE-luc), (C) NFAT/Ca2+ (NFAT-luc) and (D) GR (MMTV-luc). Cells were stimulated with ascending concentrations of Cpd1 and reference agonists: (A) 10 nM rmWnt-3a, (B) 10 µM isoproterenol, an agonist for endogenously expressed Gs-coupled β2 adrenergic receptors, (C) a combination of 100 nM phorbol 12-myristate 13-acetate (PMA) and 100 nM thapsigargin (Thaps), which activate protein kinase C and Ca2+-signaling, respectively, and (D) 1 µM of the GR agonist dexamethasone (Dex). (TIF) [file pone.0019185.s005.tif]
